# Implementation of unassisted and community-based HIV Self-Testing (HIVST) during the COVID-19 pandemic among Men-who-have-sex-with-Men (MSM) and Transgender Women (TGW): A demonstration study in Metro Manila, Philippines

**DOI:** 10.1371/journal.pone.0282644

**Published:** 2023-03-09

**Authors:** John Danvic T. Rosadiño, Ronivin G. Pagtakhan, Matthew T. Briñes, Jeanno Lorenz G. Dinglasan, Denis P. Cruz, John Oliver L. Corciega, Aeronne B. Pagtakhan, Zypher Jude G. Regencia, Emmanuel S. Baja

**Affiliations:** 1 LoveYourself Inc., Mandaluyong City, Philippines; 2 Faculty of Management and Development Studies, University of the Philippines Open University, Los Baños, Laguna, Philippines; 3 College of Medicine, Pamantasan ng Lungsod ng Maynila, Manila, Philippines; 4 Nursing Department, Centro Escolar University Makati, Makati City, Philippines; 5 Department of Clinical Epidemiology, College of Medicine, University of the Philippines Manila, Paz Mendoza Building, UPCM, Manila, Philippines; 6 Institute of Clinical Epidemiology, National Institutes of Health, University of the Philippines Manila, Manila, Philippines; University of the Witwatersrand, SOUTH AFRICA

## Abstract

**Objective:**

The study aimed to demonstrate the feasibility of an unassisted and community-based HIV self-testing (HIVST) distribution model and to evaluate its acceptability among men-having-sex-with-men (MSM) and transgender women (TGW).

**Methods:**

Our demonstration study focused on implementing the HIVST distribution model in Metro Manila, Philippines. Convenience sampling was done with the following inclusion criteria: MSM or TGW, at least 18 years old, and had no previous HIV diagnosis. Individuals taking HIV pre-exposure prophylaxis, on antiretroviral therapy, or female sex at birth were excluded. The implementation of the study was done online using a virtual assistant and a delivery system via courier due to COVID-19-related lockdowns. Feasibility was measured by the number of HIVST kits successfully delivered and utilized and the HIV point prevalence. Moreover, acceptability was evaluated by a 10-item system usability scale (SUS). HIV prevalence was estimated with linkage to care prioritized for reactive participants.

**Results:**

Out of 1,690 kits distributed, only 953 (56.4%) participants reported their results. Overall, HIV point prevalence was 9.8%, with 56 (60.2%) reactive participants linked to further testing. Furthermore, 261 (27.4%) of respondents self-reported, and 35 (13.4%) of the reactive participants were first-time testers. The HIVST service had an overall median and interquartile range (IQR) SUS score of 82.5 (IQR: 75.0, 90.0), rendering the HIVST kits very acceptable.

**Conclusions:**

Our study suggests the acceptability and feasibility of HIVST among the MSM and TGW in Metro Manila, Philippines, regardless of their age or HIV testing experience. In addition, other platforms of information dissemination and service delivery of HIVST should be explored, including access to online instructional videos and printed materials, which may facilitate easier use and interpretation of results. Furthermore, due to our study’s limited number of TGW respondents, a more targeted implementation strategy to reach the TGW population is warranted to increase their access and uptake of HIVST.

## Introduction

In 2018, the World Health Organization (WHO) recommended the use of HIV self-testing (HIVST) as an innovative approach to increase HIV testing capacity and to reach high-risk populations, particularly men-who-have-sex-with-men (MSM) and transgender women (TGW) [1]. HIVST is a WHO-recommended differentiated HIV testing option available to reach people with HIV who do not know their status and those who have previously been diagnosed with HIV but are not on antiretroviral therapy (ART) [2]. Assisted or unassisted HIVST is done through a rapid diagnostic HIV test, using either blood or oral. It is an accurate and convenient test that respects privacy and confidentiality, outweighing current barriers to facility-based testing among key populations [3, 4].

In the Philippines, an increasing trend is observed in terms of new infections, and only about 68% of people-living-with-HIV (PLHIV) knew their status last 2020 [5], a figure far from the 90-90-90 targets of the Joint United Nations Programme on HIV/AIDS (UNAIDS) [6]. An average of 29 new cases per day was recorded in January 2021. Among the newly reported cases, 89% were MSM, with 41% from the National Capital Region (NCR) [7]. Only 28% of MSM were estimated to have undergone HIV testing and were aware of their status. Accordingly, more than half (57%) of the TGW had an HIV test, where a quarter of them tested in the past 12 months. The MSM and TGW populations engage in sex at an early age (median of 16 years old), but protective behavior was practiced later (median age of first condom use at 19 years old). Condom use during the last anal sex was at 40%, and consistent condom use with the previous three anal sex partners was even low at 13%, putting them at a higher risk for HIV than other key populations [8]. As a low prevalence [9–11], and high incidence [6] country in HIV cases based on the estimates, reaching PLHIV who have unknown status has been difficult.

With the enactment of the Philippine Republic Act 11166, any person who engages in high-risk behavior below 15 years old is eligible for HIV testing with the assistance of social or healthcare workers, removing legislative barriers to testing among the young key population [12]. In addition, increasing HIV testing uptake among key populations is essential [13] since incidence cases are concentrated in these populations. The inadequate knowledge about HIV and the high stigma towards PLHIV among key populations also play a significant role in preventing access to testing and treatment [14]. Because of the current situation of the HIV epidemic in the Philippines, our study involved a multisectoral effort to implement an HIVST demonstration study among MSM and TGW in Metro Manila, Philippines. We aimed to demonstrate the feasibility of an unassisted and community-based HIVST model in terms of HIVST uptake, reporting rate, point prevalence, and linkage to confirmatory testing by distributing HIVST kits to MSM and TGW. Furthermore, we also aimed to evaluate the usability of the HIVST kits to determine their acceptability.

## Materials and methods

### Study design and setting

**Fig 1:**
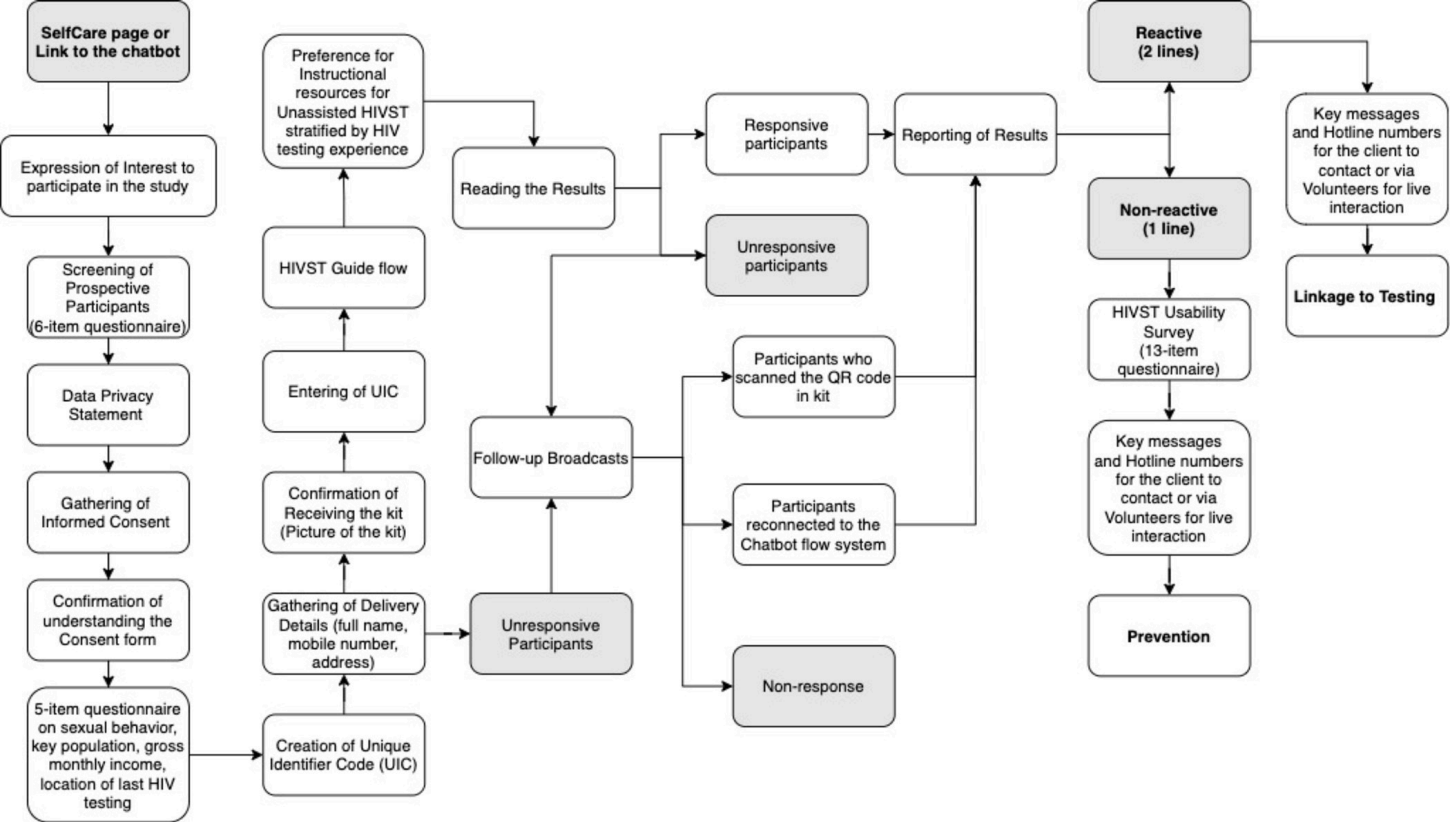
Virtual assistant flow for unassisted HIVST.

Our implementation research applied a quasi-experimental study design that focused on planning and implementing an unassisted and community-based model of HIVST service in Metro Manila, Philippines [15, 16]. Due to the limitations and lockdowns caused by the coronavirus COVID-19 pandemic, the HIVST service delivery utilized the existing online channels of The LoveYourself, Inc. (TLY). This community-based, volunteer-run organization offers HIV and other sexually-health-related services in Metro Manila, Philippines. Moreover, an automated online messaging system was designed to gather information from target participants and deliver the HIVST kits to those identified to be eligible. The online followed a five-part flow process: expression of interest, pre-qualification process, confirmation & delivery, guided HIV self-testing process, and evaluation & post-testing process (see Fig 1 for details).

**Fig 2:**
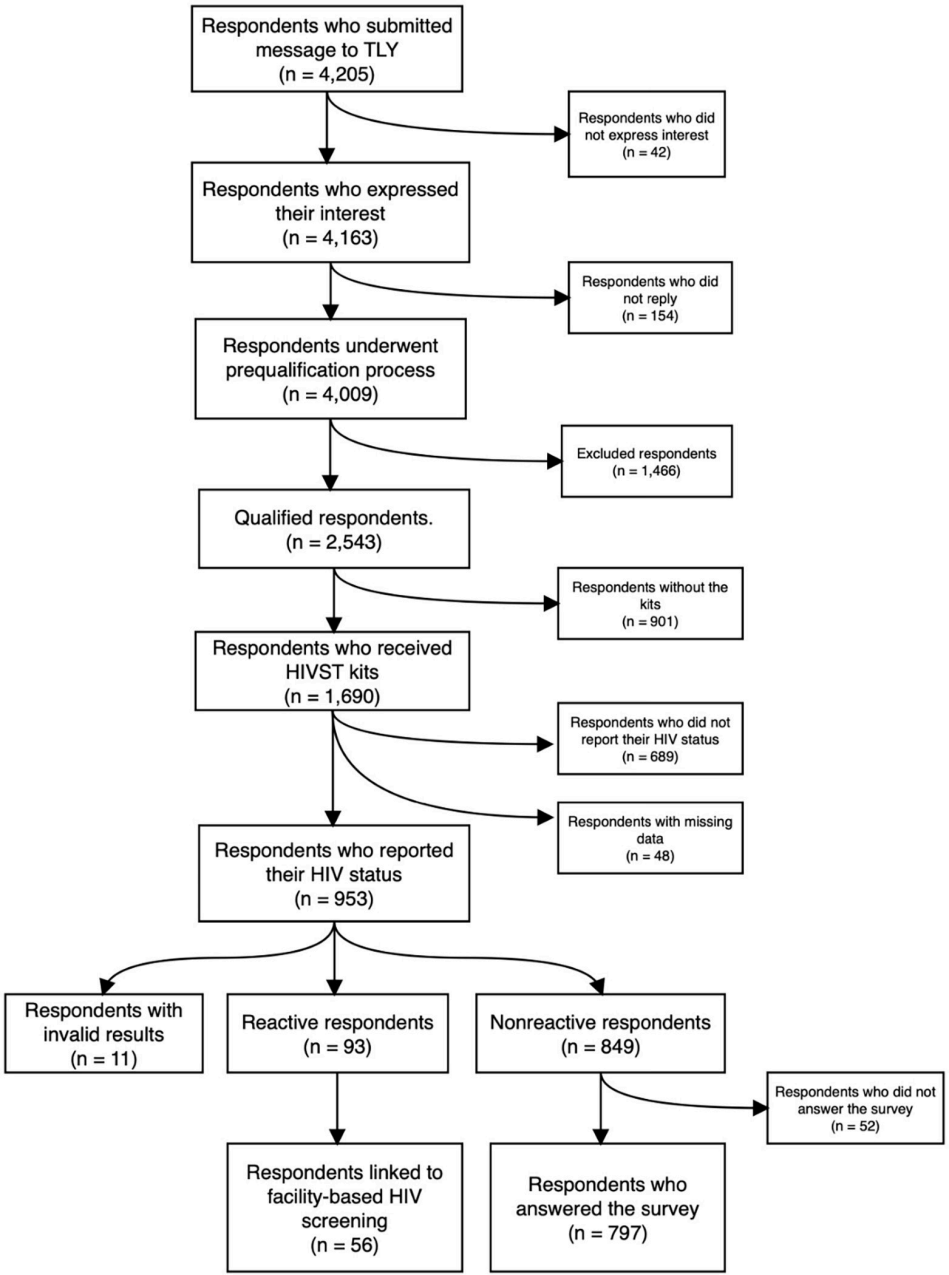
HIVST feasibility cascade chart.

The HIVST kits (SURE CHECK® HIV 1/2 Assay USA—Chembio Diagnostics Hauppauge, New York) were covered with white paper and delivered through a private courier to maintain privacy and confidentiality. Furthermore, the study team monitored and validated the number of participants who accessed the virtual assistant system. The entire demonstration study was implemented from April 2020 to May 2020. **S1 and S2 Figs, Fig 2.** provide instructions about the kit and guides the participants in using the HIVST kits.

### Study participants

During the study period, individuals in the age bracket of 18 to 49 identified themselves as MSM or TGW, had no previous diagnosis of HIV, and resided in Metro Manila were included in the study. On the other hand, individuals taking Pre-exposure Prophylaxis for HIV (PrEP), on ART, and/or female sex at birth were excluded. Following the manufacturer’s specifications, participants taking PrEP were excluded from the study as taking antiretrovirals in any form, including PrEP, could produce a false reactive result. Convenience sampling on a first-come, first-served basis was used to select the study participants.

### Data collection and outcome measurements

The study utilized location-targeted Facebook advertisements to promote the study and to invite potential target participants. In addition, the study partnered with an organization that has already been offering HIV testing, prevention, and treatment services to key populations in the country. Responses were gathered through the virtual assistant and were encrypted and automatically transported to a database in real-time. At the end of data collection, the database was anonymized by assigning a unique identifier code for each client.

The socio-demographic characteristics of the participants were collected through the virtual assistant. The feasibility of delivering HIVST kits to the target key populations in a community setting was assessed based on the following parameters: the number of HIVST kits distributed (HIVST uptake), the HIV reactivity rate, and the linkage to further testing. In addition, information on the participant’s HIV testing experience (never/ever tested for HIV) and preference on the type of sources of HIVST information was also surveyed.

Furthermore, the acceptability of the HIVST kit was the main outcome of the study. It was measured using a 10-item System Usability Scale (SUS), a standard tool that allows users to assess the usability of a given product or service [17–19]. Briefly, it is comprised of ten validated statements, which cover five positive aspects and five negative aspects of a particular tool, system, or kit. The final score is out of 100, wherein each respondent answered every question on a Likert scale from Strongly Disagree to Strongly Agree. Moreover, a SUS score of less than 50 was considered “Not Acceptable” and would imply that the HIVST kit will have usability issues. SUS scores between 50 to 70 were classified as “Marginal,” whereas a SUS score of greater than 70 was categorized as “Acceptable,” with varying degrees of usability [18]. The SUS tool was only offered to non-reactive clients through the virtual assistant. Reactive clients were excluded due to possible high emotional stress from the result of using the HIVST kit, which may affect their response to the survey.

Moreover, counselors assigned to each client discussed evidence-based preventive strategies such as using PrEP to non-reactive clients to decrease their risk of acquiring HIV and other sexually transmitted infections. Furthermore, counselors prioritized linkage to confirmatory testing for the reactive clients and emphasized the importance of early initiation of treatment. The reactive clients were referred to “acXess by LoveYourself,” the ambulatory service created by the TLY team to overcome the mobility restrictions caused by the announcement of enhanced community quarantine lockdowns to control the spread of COVID-19. The research team excluded the reactive group from answering the survey to prioritize confirmatory testing and treatment referral. In addition, the research team contacted the participants who received invalid results and were given instructions to visit the nearest HIV facility for immediate retesting. Lastly, the research team sent notifications via the virtual assistant to those who did not disclose their HIVST result to remind them to report their test result.

### Data analysis

Descriptive statistics were calculated to describe the participant’s characteristics using the mean and standard deviation or median and interquartile range (IQR) for continuous variables and frequencies and percentages for categorical variables. SUS calculation was carried out using the formula below:

$$SUS=\frac{1}{n}\sum_{i=1}^{n}\mathrm{norm}\sum_{j=1}^{m}\{\begin{array}{ll} q_{ij}-1 & \mathrm{for}\;Q_{j}\;mod2>0\\ 5-q_{ij} & \mathrm{otherwise} \end{array}$$ [Eq 1]

where n = number of subjects, m = 10 (number of questions), *q*_*ij*_ = score for question *j* for subject *i*, norm = 2.5, and *Q*_*j*_ = question number [20].

Chi-square test and Fisher’s Exact test were used to determine relationships between the preference of various instructional resources for HIVST and the HIV testing experience and age category of the participants. In addition, a permutation test was used to assess if the SUS scores stratified by age, gender identity, and HIV testing experience were significantly different within the stratified participant characteristics. All statistical analyses were carried out using STATA 17 (www.stata.com) software. A *p*-value of ≤ 0.05 was considered statistically significant.

### Ethical considerations

The University of the Philippines Manila Research Ethics Board (UPMREB) has approved the ethical clearance of this study with UPMREB CODE: 2019-474-01. All data were treated following the Philippine Data Privacy Act of 2012 (Republic Act 10173). Informed consent forms signed online by the participants were collected from the start of the study.

## Results

### HIVST cascade

The cascade of the feasibility of delivering HIVST kits to the target participants is presented in Fig 2. These numbers were combined data from the virtual assistant analytics generated at the end of the study period. Out of 4,205 respondents who submitted a message, 4,163 (99.0%) respondents expressed their interest in getting the HIVST kit, and 4,009 (95.3%) respondents underwent the pre-qualification process, but only 2,543 (60.5%) respondents were eligible to receive the kit. Furthermore, respondents with multiple entries were removed, and 2,232 (53.1%) unique respondents remained to receive the HIVST kits. In addition, 542 qualified respondents did not receive their kit due to limited mobility and restrictions imposed by the COVID-19 lockdown. (see S1 Table for the study characteristics of the qualified respondents).

Moreover, 1,690 HIVST kits were successfully distributed to respondents, but only 953 participants reported their results, a reporting rate of 56.4%. The observed HIV point prevalence from April 2020 to May 2020 using the HIVST kits was 9.8% (93/953). Furthermore, 60.2% (56/93) were linked to further testing among the reactive participants. Additionally, non-reactive participants were asked to answer the SUS survey, and 93.9% (797/849) sent their responses. Characteristics of the respondents who received the kits, who reported their HIV results, and who answered the survey are presented in Table 1.

**Table 1:** Characteristics of the study population.

Characteristics ^a^	Received the	Reported the results	Answered the
	HIVST kit	of the HIV test	online survey
	(n = 1,690)	(n = 953)	(n = 797)
Age [mean (SD^b^)]	28.1 (5.3)	27.9 (5.2)	28.0 (5.3)
18 to 24 years old	452 (26.7)	277 (29.1)	231 (29.0)
25 to 34 years old	984 (58.2)	559 (58.6)	459 (57.6)
35 years old & above	204 (12.1)	115 (12.1)	105 (13.2)
Missing data	50 (3.0)	2 (0.2)	2 (0.2)
Gender identity			
MSM	1,531 (90.6)	892 (93.6)	744 (93.4)
TGW	38 (2.2)	19 (2.0)	16 (2.0)
Prefer not to say	70 (4.2)	40 (4.2)	35 (4.4)
Missing data	51 (3.0)	2 (0.2)	2 (0.2)
Previous testing experience			
Got tested	1,187 (70.2)	692 (72.6)	585 (73.4)
Never tested	454 (26.9)	261 (27.4)	212 (26.6)
Missing data	49 (2.9)	-	-
Testing site commonly used			
TLY	731 (43.3)	426 (44.7)	372 (46.7)
Social Hygiene Clinics	144 (8.5)	79 (8.3)	68 (8.5)
Private Clinic	91 (5.4)	50 (5.2)	43 (5.4)
Hospital (Private/Public)	114 (6.7)	65 (6.8)	48 (6.0)
CBS Motivator	25 (1.5)	17 (1.8)	16 (2.0)
Other centers	82 (4.9)	55 (5.8)	38 (4.8)
Never tested	454 (26.9)	261 (27.4)	212 (26.6)
Missing data	49 (2.9)	-	-
Monthly Income, PHP			
< 10,000	144 (8.5)	75 (7.9)	53 (6.7)
10,000–19,999	377 (22.3)	198 (20.8)	162 (20.3)
20,000–29,999	338 (20.0)	202 (21.2)	173 (21.7)
30,000–49,999	236 (14.0)	142 (14.9)	127 (15.9)
50,000–99,999	99 (5.9)	58 (6.1)	49 (6.2)
100,000 and up	43 (2.5)	27 (2.8)	23 (2.9)
Not applicable ^c^	175 (10.4)	114 (11.9)	99 (12.4)
Prefer not to say	229 (13.6)	137 (14.4)	111 (13.9)
Missing data	49 (2.9)	-	-
HIV test result			
Reactive	-	93 (9.8)	-
Non-reactive	-	849 (89.1)	797 (100.0)
Invalid	-	11 (1.1)	-
SUS score [median (IQR^d^)]	-	-	82.5 (75.0, 90.0)

^a^ Distributions of variables are reported as n (%) unless specified otherwise

^b^ SD: Standard deviation

^c^ Includes students and unemployed

^d^ IQR: Interquartile range.

In Table 1, the mean age of the respondents who received the kit, who reported the HIV result, and who answered the survey was similar (~28 years old). Most of them were MSM (~93%) across all groups, and only about ~2% were TGW. Moreover, 1 out of 5 respondents had an average monthly income between PhP 10,000–19,999 (~USD 200–399.98), while almost 3% of them earned ≥ PHP 100,000 (~USD 2,000) monthly. Furthermore, the overall SUS median score was 82.5 (IQR: 75.0, 90.0), making the HIVST kit very usable and acceptable.

Of the 56 reactive participants linked to further testing, approximately 2 out of 3 belonged to the 25 to 34 age group (66.1%), and 19 out of 20 were MSM (94.6%). Moreover, among the 261 first-time testers, the estimated HIV point prevalence was 13.4% (35/261) (see Table 2 for details).

**Table 2:** Characteristics of reactive respondents linked to further testing (n = 56).

Characteristics ^a^	Values
Age groups (years)	
18 to 24	17 (30.3)
25 to 34	37 (66.1)
35 years old & above	2 (3.6)
Gender Identity	
MSM	53 (94.6)
TGW	0 (0.0)
Preferred not to say	3 (5.4)
Testing Experience	
Never Tested	35 (62.5)
Ever Tested	21 (37.5)

^a^ Distributions of variables are reported as n (%).

The respondents’ preferences when it comes to sources of instructional materials on HIVST were also collected and summarized. More than half of the participants preferred to use instructional videos (51.9%) and printed materials or inserts (52.4%). This preference is true regardless of the HIV testing experience of the respondents. In contrast, a landline hotline option was the least preferred among all types of resources (3.2%). In addition, no difference in the preference for various sources of instructional materials was observed between ever-tested and never-tested participants (*p*-values > 0.05) (see Table 3 for details). Aside from the data presented in Table 3, we also looked at the respondents’ preferences for instructional materials stratified according to age. Results also showed some significant difference recorded across all age groups. Participants belonging to the 35 and above age bracket were slightly significantly different from other age groups in preferring printed materials as instructional materials (see S2 Table for details).

**Table 3:** Preference for instructional resources stratified by HIV testing experience (N = 797).

Resources	Total		Ever Tested		Never Tested		*p*-values^a^
	n = 792		n = 584		n = 208		
	n %		n %		n %		
**Instructional videos (%)**							0.33
Yes	411	51.9	297	50.9	114	54.8	
No	381	48.1	287	49.1	94	45.2	
Missing data	5						
**Printed Materials/Inserts (%)**							0.63
Yes	415	52.4	309	52.9	106	51.0	
No	377	47.6	275	47.1	102	49.0	
Missing data	5						
**Talking with celebrity endorsers via Messenger (%)**							1.00
Yes	145	18.3	107	18.3	38	18.3	
No	647	81.7	477	81.7	170	81.7	
Missing data	5						
**Hotline (%)**							0.11
Yes	25	3.2	15	2.6	10	4.8	
No	767	96.8	569	97.4	198	95.2	
Missing data	5						

^a^ Chi-square test

### HIVST Kit System Usability Scale (SUS) score

The HIVST kit used in the demonstration study had an overall SUS median score (IQR) of 82.5 (IQR: 75.0, 90.0). The SUS score of the participants was further stratified based on age group, gender identity, and HIV testing experience. The SUS median scores differ significantly among the three age groups (*p-*value < 0.01), with the 35 to 49 age group having the highest SUS median score (85.0; IQR: 77.5, 92.5) and the 15 to 24 age group having the lowest SUS median score (80.0; IQR: 72.5, 87.5). For gender identity, both the MSM & TGW participants had the same higher SUS median score (82.5; IQR: 75.0, 90.0) compared to the other participants who preferred not to disclose their gender identity. However, the SUS median scores were not significantly different (*p-value* = 0.25). Lastly, participants with prior HIV testing experience had a higher SUS median score than HIV never-testers; the SUS median score of the two groups was significantly different (*p-value* = 0.04). All the observed SUS median scores were under the “acceptable” category range, rendering the HIVST kit usable regardless of age category, gender identity, and HIV testing experience (see Table 4 for more details).

**Table 4:** HIVST kit SUS scores, stratified by non-reactive participants’ characteristics (N = 797).

Participants stratified by characteristics	n	Usability Scores Median [IQR]^a^	*p*-values^b^
Overall	783	82.5 [75.0, 90.0]	
Missing data	14		
Age groups (years)			
15 to 24	225	80.0 [72.5, 87.5]	<0.01
25 to 34	451	82.5 [75.0, 90.0]	
35 years old & above	105	85.0 [77.5, 92.5]	
Missing	16		
Gender Identity			
MSM	731	82.5 [75.0, 90.0]	0.25
TGW	15	82.5 [75.0, 90.0]	
Preferred not to say	35	80.0 [70.0, 85.0]	
Missing data	16		
HIV Testing Experience			
Never Tested	206	80.0 [72.5, 90.0]	0.04
Ever Tested	577	82.5 [75.0, 90.0]	
Missing data	14		

^a^ IQR: Interquartile range

^b^ Permutation test

## Discussion

Based on the number of individuals who expressed their interest in getting the HIVST kit, there was an apparent demand for an alternative method to HIV testing. There were 2,235 eligible to receive the HIVST kit; 1,690 participants received their kit with a reporting rate of 56.4% and an HIV point prevalence of 9.8%. The non-reactive participants who responded to the survey gave the kit an overall SUS median score of 82.5, making it acceptable and easy to use for the MSM and TGW at-risk populations. In addition, participants preferred to watch instructional videos and printed materials than call a hotline to use the kit. The usability of the kit was also dependent on the user’s age, as seen through the higher SUS score in the 35 to 49 age group compared with the lower age groups. Participants with previous HIV testing experience also gave a higher SUS score than first-time HIV testers.

Innovative ways of reaching the MSM and TWG populations in the country for testing and educating, especially during a pandemic, is an effective strategy for controlling the large-scale generalized HIV epidemic among these high-risk groups. Confounding factors such as low condom use, unsafe injecting drug use practices, lack of sexual health education and misconception about HIV/AIDS, and religion and conservatism further increase the rate of infection in the country [21]. In the Philippines, the fear of testing positive prevents access to HIV testing, and some would prefer to die than face judgment from other people. In a study in Singapore, HIVST’s advantage of being completely anonymous, ease of access, and autonomy, made it preferable, with an inclination towards blood-based testing. However, the participants did not feel confident in using blood-based testing due to fear of pricking themselves, which they felt could affect the result [22].

In our study, anonymity and confidentiality generated a high demand for the HIVST kit, with more than 4,000 expressing interest. There was a recorded 56.4% reporting rate and a 9.8% reactivity rate among those eligible to receive the kit. The majority of those reported were in the 25–35 age group and with testing experience. Other studies on the feasibility and acceptability of HIVST kits were conducted in Africa [23–27] and elsewhere [28–30]. In a study done in Africa, they compared the usability of the oral and fingerstick methods of HIVST. The participants reported the kits to be easy to use, especially those with previous testing experience. The inclusion of visual instructions such as videos or photos and simple sample collection reduced errors in using the kits, especially in interpreting results. They also found the oral test easier to use but preferred the fingerstick test, which may improve with further use and instructional resources [24, 25]. Following the findings in previous studies [25, 31, 32], instructional materials were included in our HIVST package. This inclusion of instructional materials made the kit even more accessible as this facilitated learning to use the kit easier. In addition, our participants preferred to access the instructional videos found online to guide them in using the kit and interpreting the results. However, in a limited resource setting country like the Philippines, internet access could still be a challenge in some areas, which made the second preference of instruction to use was the printed materials/inserts.

The median ages of participants who took HIVST from other studies [30, 33, 34] were found to be in agreement with our study. In addition, the kit’s usability score and the user’s age were significantly related, with the observed highest usability score among the 35 to 49 age group compared with the other age groups, which could be indicative of the comfortability and confidence of the older age group in using the kits. Our result could also be meaningful for getting the HIVST kit. The younger age group’s purpose could primarily be exploration and curiosity regarding the self-testing kit, whereas the older age group, more critical, could mostly be health-related.

Implementing HIVST in a community-based setting [30, 33–37] in partnership with different stakeholders is a common feature across studies on HIVST conducted online [30, 33, 34] since reactive participants can be further linked to testing and treatment easily, reflecting the central goal of HIVST [1, 38]. A review article summarized the implementation outcomes for HIVST that would help implementers assess the program’s success. They found HIVST acceptable and appropriate, perceived convenient, and better at maintaining confidentiality than standard testing. However, the usual concerns were the lack of counseling and linkage to care, the occurrence of user errors, and cost-effectivity [23].

Our demonstration study maximized the usage of the virtual assistant, from ordering the kit to reporting the results and linking the reactive participants to confirmatory testing and treatment. In addition, video materials were included in the system for more HIV treatment and prevention information. Moreover, the kit consists of instructions for use, including a guide on how to use the kit and contact details to ensure participants are well-guided on their next steps should they get a reactive result. The uptake of HIVST kits, HIV point prevalence, number of first-time testers, and usability of the HIVST kit were the leading indicators for the success of our study implementation. Our study distributed a significant number of kits across the target populations, which resulted in a higher finding on point prevalence. The first-time testers who reported their results in our study were similar to another research finding [33]. Furthermore, the primary goal of our study was to provide evidence that HIVST can capture first-time testers among members of the key populations (MSM and TGW) who have undiagnosed HIV. In addition, the results of our HIVST implementation suggest feasibility in the Philippines.

Association studies linking the different socio-demographic variables and sexual behaviors and the HIV status of the participants were mainly seen in other feasibility and acceptability researches on HIVST kits [33, 34, 39–41]. One study reported that among first-time testers who had reactive results, having multiple sexual partners and reporting inconsistent condom use were associated with an HIV-positive result [33]. In contrast, our study did not require reactive participants to answer questions on HIVST usability because linking them to further confirmatory testing and treatment was more prioritized than getting insights from the reactive participants. Therefore, further studies may be needed to look at the predictors of HIVST among reactive first-time testers in the Philippines, particularly the MSM and TGW populations, as supported by our results.

The study also included the TGW population since they are also considered a high-risk group in the Philippines for acquiring HIV. We reached this target population but only captured a modest sample size. This finding is an important observation since TGWs were considered high-risk for HIV, with a high global pool prevalence of HIV (17.7%) among TGW in low- and middle-income countries [42]. This high global pool prevalence of HIV is evidenced by the high transmission probability of unprotected receptive anal intercourse consistently engaged by TGW, like most MSM [43, 44], in addition to consistently rare access to appropriate clinical prevention, treatment, or care services specifically in low- and middle-income countries [45, 46]. Nevertheless, our study provided data regarding the uptake of HIVST among TGW. Future research must focus on the design to disseminate demand creation strategies for TGW and increase their participation in the HIVST to generate evidence relevant to their community.

In terms of acceptability, HIVST kit usage in other studies was usable across the key populations, particularly on MSM and TGW [4, 40, 41, 47, 48], which is similar to the findings from our study. In addition, reports on social harm were not observed during the implementation of our study, a result comparable to other studies [49–51], while other studies reported otherwise [52–56]. However, stigma still plays a role in HIV diagnosis in the Philippines, and self-harm could still be possible after receiving a diagnosis. Partnering with organizations and other institutions that provide counseling and quick linkage to care and treatment would help prevent self-harm. In our study, the participants were given options to contact a center using the virtual assistant and the materials included in the kit.

HIVST offers a discreet, convenient, and empowering way for those who may not otherwise test in a facility [49]. The findings of our study showed that HIVST is effective in reaching those who want to know their HIV status. Since the low uptake of HIVST was observed among the young key population and first-time testers in our study, an opportunity for future implementers to capitalize on creating materials for HIVST targeted at these populations is warranted. Key messages must be different depending on the client’s age bracket and HIV testing experience. For clinicians, our study provided evidence regarding the demand, uptake, and acceptability of HIVST among MSM and TGW in the Philippines that can help them be informed in deciding how to offer HIVST to their patients. For implementers, the design of our demonstration study can be patterned to future programs on HIVST that are tailored to other local MSM and TGW communities in the Philippines. HIVST is said to increase HIV testing [49, 57, 58] among key populations; thus, implementers should include HIVST as one of the key indicators in their existing HIV Testing and Counselling Services (HTS). Lastly, funding agencies or companies may consider HIVST their Corporate Social Responsibility (CSR) service by helping the community as a potential sustainable funding source [59]. Some countries in Africa scaled up HIVST distribution through catalytic donor investments [60, 61].

The delivery of HIVST kits to the MSM and TGW populations is feasible as reflected by high HIVST kit uptake, reporting rate, point prevalence, and reactive participants linked to further testing. Most reactive participants were linked to testing and treatment through TLY, while three were linked to other treatment hubs. TLY has testing and treatment facilities in key locations in the metro, which made the recruitment process for this study easier. Moreover, participants who tested reactive were immediately linked to testing and treatment with the help of volunteer TLY counselors, decreasing the cost of the study for post-counseling services. Using a virtual assistant to deliver HIVST kits during the COVID-19 pandemic, the research team further protected the privacy and confidentiality of interested respondents and participants. Various options on how to access instructional materials to use the HIVST kit were also provided (e.g., through the virtual assistants or the materials included with the kit), ensuring participants’ autonomy.

Limitations were identified in the implementation of our study. Mobility restrictions decreased the number of HIVST kits delivered; Metro Manila was inaccessible due to strict lockdowns imposed by the government during the COVID-19 pandemic. In addition, convenience sampling of qualified participants was done due to the high demand for the HIVST kit, which could result in a potential selection bias creating a false equivalence of the data. Selected participants may have a higher desire to use the HIVST kit the moment they sign up and order it online, which may shift the calculated usability of the HIVST kits away from the null. Conversely, the false equivalence could also shift the calculated usability of the HIVST kit towards the null when sampling fails to reach the intended target population. Moreover, only MSM and TGW participants with internet connections within Metro Manila could receive the HIVST kits.

Furthermore, some participants did not report their results even if a follow-up was done. However, the distribution of the respondent’s characteristics who received the kit, who reported their HIV result, and who answered the survey did not change at each stage of the HIVST cascade. This similar distribution implies that the respondents who answered the survey may be considered representative of the study’s intended target population. The selection of non-reactive participants to answer the SUS limits the generalizability of our results. The non-reactive participants may have an overly positive response due to getting a non-reactive result, which may lead to an increase in the SUS scores. In addition, our results did not include reactive respondents. Hence, the findings may not represent the target MSM or TGW who are HIV-reactive. Moreover, our study findings cannot be generalizable to other high-risk and at-risk populations. Nonetheless, more studies are needed to validate the results of our research.

Future research may benefit from our study. Additional items on demographic questionnaires and questions on condom use and sexual behaviors can be included to characterize further those who want to use the HIVST kit. Moreover, barriers and facilitators considered in accessing HIV testing and treatment must also be ascertained among participants since these could potentially help design HIVST programs effectively and efficiently in the future. In addition, future qualitative studies may also include insights from the non-respondents on HIVST and the difficulty or ease of HIVST usage, as this information may provide a valuable contribution to the future nationwide implementation of the HIVST program. Finally, in distributing HIVST kits to clients, implementers can explore other means of distributing kits through pharmacies, social hygiene clinics, etc., to reach those who do not have internet access [62, 63].

As we increase HTS coverage, the proportion of HIV-positive diagnoses may decrease across all approaches, necessitating a more strategic and focused method to achieve consistent or higher levels of positivity for all HTS approaches, including HIVST [59]. HIVST can also contribute to a more comprehensive provider-initiated HTS in public clinics that are highly congested with a limited testing capacity or poorly managed [64, 65].

## Conclusion

Introducing new HIV testing modalities, such as HIVST, is vital to reach more people to know their HIV status and access treatment or prevention packages. Our demonstration study showed that the implementation of HIVST in Metro Manila is feasible. In addition, the HIVST kits used were generally acceptable to the MSM and TGW populations regardless of age or HIV testing experience. Instructional materials and videos would further help ensure the correct use of the kits. Implementers should explore the strengthening and utilization of other platforms to reach more at-risk populations. In a limited resource country like the Philippines, stable internet connectivity can be a challenge in accessing this HIVST service, especially during a pandemic. Moreover, due to the low turnout of TGW in this study, more targeted messaging should be done to ensure further reach. This study can serve as a guide in overcoming possible barriers to this innovation by enabling the national government to create national standardized guidelines on HIVST implementation in the country.

## Supporting information

S1 Fig Instructions for use (HIV Self-Testing Kit) part 1. (TIFF)

S2 Fig Instructions for use (HIV Self-Testing Kit) part 2. (TIFF)

S1 Table Characteristics of the qualified participants to receive the kits, stratified according to HIV testing experience (n = 2,232). (PDF)

S2 Table Preference of instructional resources stratified by age (n = 797). ^a^Chi-square test; ^b^Fisher’s exact test.
(PDF)
