# Supplementary figures and images for: Implementation of unassisted and community-based HIV Self-Testing (HIVST) during the COVID-19 pandemic among Men-who-have-sex-with-Men (MSM) and Transgender Women (TGW): A demonstration study in Metro Manila, Philippines
Source: PLoS One. 2023 Mar 9;18(3):e0282644. doi: 10.1371/journal.pone.0282644 (PMC9997871; doi:10.1371/journal.pone.0282644)

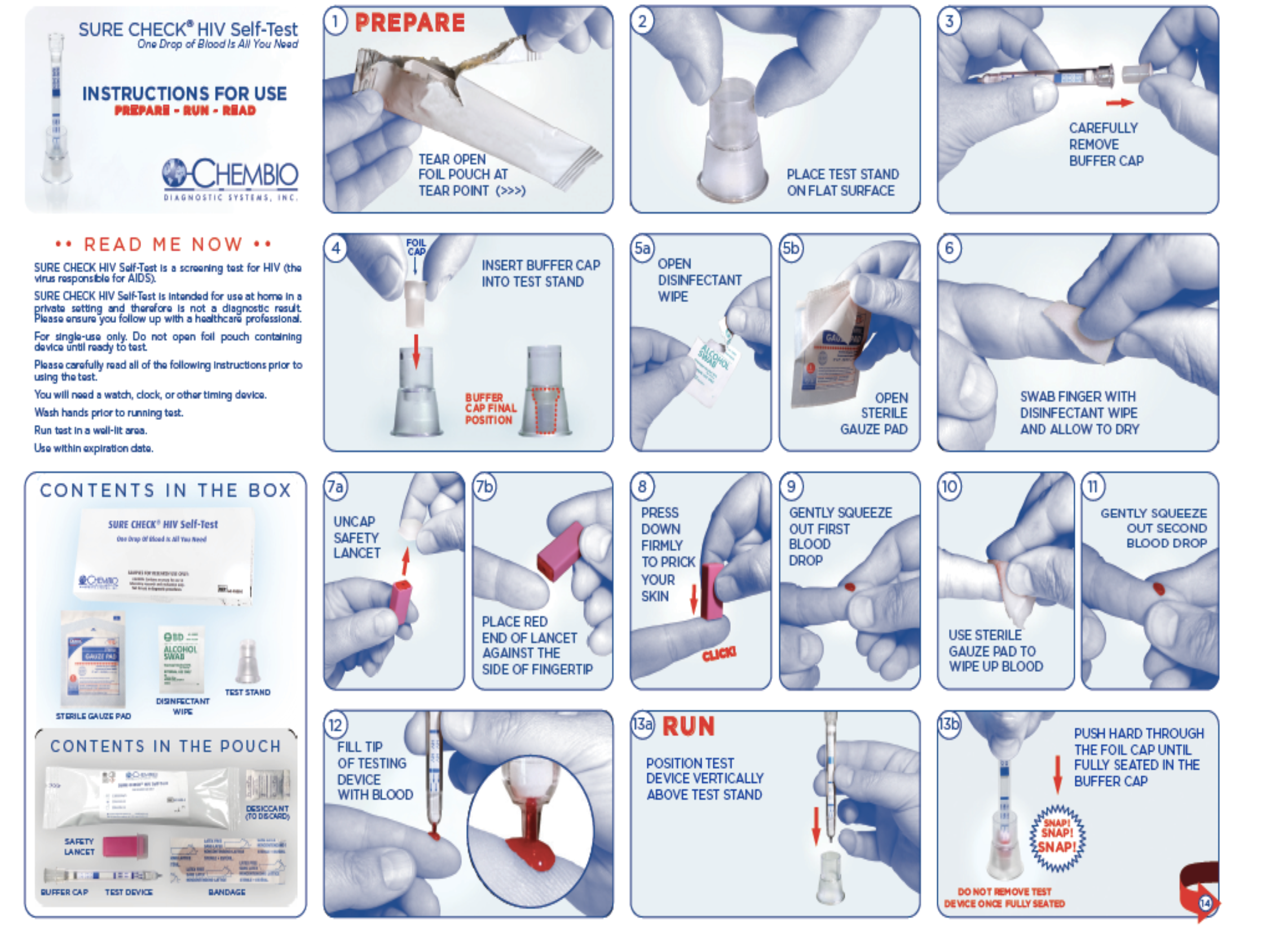

Supplement: S1 Fig — (TIFF) [file pone.0282644.s001.tiff]

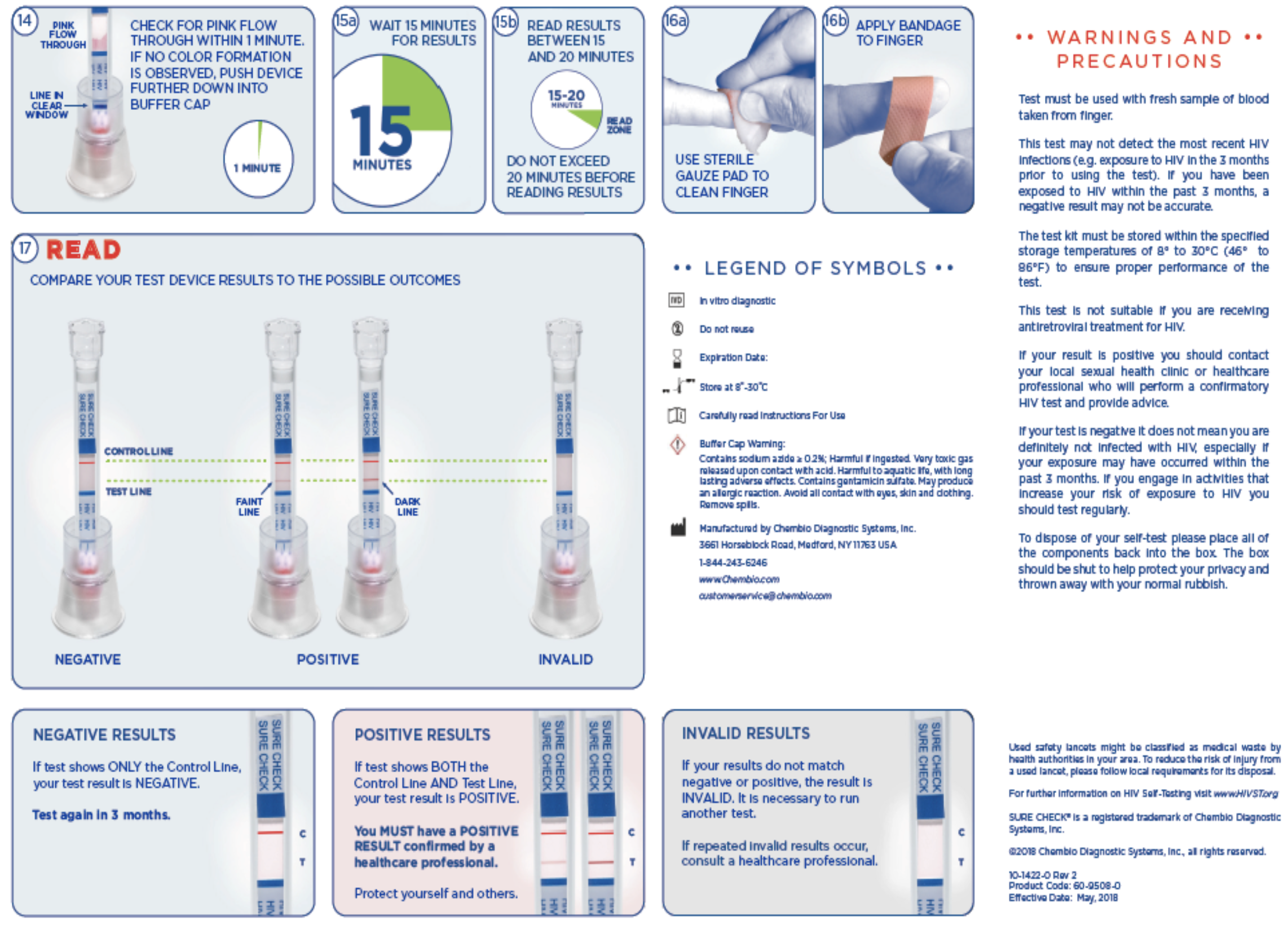

Supplement: S2 Fig — (TIFF) [file pone.0282644.s002.tiff]
