# Supplementary material for: Implementation of unassisted and community-based HIV Self-Testing (HIVST) during the COVID-19 pandemic among Men-who-have-sex-with-Men (MSM) and Transgender Women (TGW): A demonstration study in Metro Manila, Philippines
Source: PLoS One. 2023 Mar 9;18(3):e0282644. doi: 10.1371/journal.pone.0282644 (PMC9997871; doi:10.1371/journal.pone.0282644)
Supplement: S1 Table — (PDF) [file pone.0282644.s003.pdf]

Age Groups (years)	Gender Identity	HIV Testing Experience			
		With		Without	
		n	%	n	%
**18-24**	MSM	248	96.5	121	85.2
	TGW	3	1.2	10	7.0
	Undisclosed	6	0.6	11	7.7
	**Sub-total**	257	11.5	142	6.4
**25-34**	MSM	989	93.8	363	89.4
	TGW	20	1.9	16	3.9
	Undisclosed	45	4.3	27	6.7
	**Sub-total**	1,054	47.2	406	18.2
**35 and above**	MSM	266	91.4	74	90.2
	TGW	11	3.8	4	4.9
	Undisclosed	14	4.8	4	4.9
	**Sub-total**	291	13.0	82	3.7
