# Supplementary material for: Implementation of unassisted and community-based HIV Self-Testing (HIVST) during the COVID-19 pandemic among Men-who-have-sex-with-Men (MSM) and Transgender Women (TGW): A demonstration study in Metro Manila, Philippines
Source: PLoS One. 2023 Mar 9;18(3):e0282644. doi: 10.1371/journal.pone.0282644 (PMC9997871; doi:10.1371/journal.pone.0282644)
Supplement: S2 Table — aChi-square test; bFisher’s exact test. (PDF) [file pone.0282644.s004.pdf]

Resources	Total n = 797		15 – 24 years old n = 230		25 – 34 years old n = 455		35 years old & above n = 105		*p*-values
	n	%	n	%	n	%	n	%	
**Instructional videos**									0.21[a]
Yes	409	51.8	127	55.2	235	51.6	47	44.8	
No	381	48.2	103	44.8	220	48.4	58	55.2	
Missing data	7								
**Printed Materials/Inserts**									0.053[a]
Yes	413	52.3	108	47.0	241	53.0	64	61.0	
No	377	47.7	122	53.0	214	47.0	41	39.0	
Missing data	7								
**Talking with celebrity endorsers via Messenger**									0.23[a]
Yes	144	18.2	46	20.0	85	18.7	13	18.3	
No	646	81.8	184	80.0	370	81.3	92	81.7	
Missing data	7								
**Hotline**									0.001[b]
Yes	24	3.0	12	5.2	10	2.2	12	1.9	
No	766	97.0	218	94.8	445	97.8	103	98.1	
Missing data	7								
